# MiR-29a-deficiency causes thickening of the basilar membrane and age-related hearing loss by upregulating collagen IV and laminin

**DOI:** 10.3389/fncel.2023.1191740

**Published:** 2023-05-18

**Authors:** Peng Ma, Shuli Wang, Ruishuang Geng, Yongfeng Gong, Mulan Li, Daoli Xie, Yaning Dong, Tihua Zheng, Bo Li, Tong Zhao, Qingyin Zheng

**Affiliations:** ^1^School of Basic Medicine, Qingdao University, Qingdao, China; ^2^School of Basic Medicine, Binzhou Medical University, Yantai, China; ^3^Department of Hearing and Speech Rehabilitation, School of Special Education, Binzhou Medical University, Yantai, China; ^4^Department of Otolaryngology, Case Western Reserve University, Cleveland, OH, United States

**Keywords:** hearing loss, miR-29a, hair cells, basilar membrane, collagen IV

## Abstract

Age-related hearing loss (ARHL) is the most common sensory degenerative disease and can significantly impact the quality of life in elderly people. A previous study using GeneChip miRNA microarray assays showed that the expression of miR-29a changes with age, however, its role in hearing loss is still unclear. In this study, we characterized the cochlear phenotype of miR-29a knockout (*miR-29a^–/–^*) mice and found that miR-29a-deficient mice had a rapid progressive elevation of the hearing threshold from 2 to 5 months of age compared with littermate controls as measured by the auditory brainstem response. Stereocilia degeneration, hair cell loss and abnormal stria vascularis (SV) were observed in *miR-29a^–/–^* mice at 4 months of age. Transcriptome sequencing results showed elevated extracellular matrix (ECM) gene expression in *miR-29a^–/–^* mice. Both Gene Ontology (GO) annotation and Kyoto Encyclopedia of Genes and Genomes (KEGG) pathway enrichment analysis revealed that the key differences were closely related to ECM. Further examination with a transmission electron microscope showed thickening of the basilar membrane in the cochlea of *miR-29a^–/–^* mice. Five Col4a genes (*Col4a1-a5*) and two laminin genes (*Lamb2 and Lamc1*) were validated as miR-29a direct targets by dual luciferase assays and miR-29a inhibition assays with a miR-29a inhibitor. Consistent with the target gene validation results, the expression of these genes was significantly increased in the cochlea of *miR-29a^–/–^* mice, as shown by RT-PCR and Western blot. These findings suggest that miR-29a plays an important role in maintaining cochlear structure and function by regulating the expression of collagen and laminin and that the disturbance of its expression could be a cause of progressive hearing loss.

## Introduction

Age-related hearing loss (ARHL), or presbycusis, is a degenerative disease in aging mammals and is the most common sensory disorder in aging individuals (Liu and Yan, 2007; Someya and Prolla, 2010; Someya et al., 2010; Sun et al., 2022). It manifests as an age-related progressive decline in auditory function. With the aging of the population, the incidence of ARHL is increasing. ARHL is associated with communication difficulties and cognitive decline, which can lead to anxiety and depression (Kim and Chung, 2019; He et al., 2021).

The main pathological changes of ARHL are characterized by the irreversible loss of hair cells and degeneration of spiral ganglion neurons and SV in the cochlea. Many studies have shown that oxidative damage caused by excessive accumulation of reactive oxygen species (ROS) is an important intrinsic factor causing ARHL in the elderly (Tavanai and Mohammadkhani, 2017; Lyu et al., 2020; Rousset et al., 2020). Although the specific etiology of ARHL is not fully understood (He et al., 2020), it may be influenced by genetic factors and environmental factors (Peng L. et al., 2022). The ARHL phenotype can be directly mimicked by the selective knockout gene in mice. With the help of mouse mutants, the mechanism of involvement of *ahl* locus near the cadherin 23 gene and *Tmc1* locus in Beethoven mutant in ARHL has been illustrated (Noben-Trauth et al., 2003; Noguchi et al., 2006).

In addition, gene mutation in non-coding microRNAs (miRNAs) can also cause hearing loss. Indeed, many miRNAs have been characterized to affect the inner ear development of mice (Jiang et al., 2022). miR-96 is the first microRNA discovered to be associated with progressive hearing loss in humans and mice (Mencia et al., 2009; Solda et al., 2012). Its role in hair cell development has been confirmed in a mutant mouse model with a single base change in the seed region of miR-96 (Lewis et al., 2009).

The miR-29 family is a key regulator of many biological processes (Horita et al., 2021; Peng D. W. et al., 2022), and the most widely known functions include the promotion of apoptosis and the regulation of cell differentiation (Guo et al., 2018; Han et al., 2018; Peng et al., 2018). In addition, the miR-29 family may suppress many extracellular matrix (ECM) genes, including those encoding collagen, elastin, and fibrillin (Hubmacher and Apte, 2013). This has been reported in many tissues, such as neurons, nasopharyngeal carcinoma, and aortic vessels (Boon et al., 2011; Qiu et al., 2015; Ma et al., 2020). A previous study using GeneChip miRNA microarray assays showed that the expression of miR-29a in the mouse cochlea changed with age (Zhang et al., 2013), however, nothing is known about its role in auditory function and its molecular mechanisms.

In this study, our experiments using miR-29a knockout (KO) mice revealed that miR-29a was expressed in the cochlea, and miR-29a KO mice exhibited progressive hearing loss. Transcriptome sequencing results showed elevated ECM gene expression in KO mice. Both Gene Ontology (GO) annotation and Kyoto Encyclopedia of Genes and Genomes (KEGG) pathway enrichment analysis revealed that the key differences were closely related to ECM. Thickening of the basilar membrane was observed by transmission electron microscopy, which could be explained by increased levels of collagen types IV and laminin that were further validated as target genes of miR-29a in our experiments. These results suggest that miR-29a plays an essential role in maintaining cochlear structure and function.

## Materials and methods

### Animals

The miR-29a-deficient mouse strain on the C57BL/6 background was obtained from Washington University in St. Louis (Papadopoulou et al., 2011; Dooley et al., 2017). Mice were housed in a controlled environment characterized by a 12 h light/dark cycle, temperature of 22 ± 1°C, and 40–60% humidity with free access to chow and water. All mice were maintained in specific pathogen-free facilities. The animal study was reviewed and approved by the Animal Use and Care Committee of Binzhou Medical University.

Genotyping was determined by PCR of mouse tail or toe DNA. For the wild-type (WT) allele, the primer FW (5′-TTAGTCAACCACCGTTAGAA-3′) and primer RV (5′- ATTGACTGGTCAGTCATTGG-3′) were used to produce a 480 bp band. For the knockout allele, the primer FW (5′-CAGAAAGCGAAGGAGCAAAG-3′) and primer RV (RV: 5′-AATGGTTCAAACGCTCCAC-3′) were used to produce a band of 717 bp. Heterozygous mice were used for breeding to generate pups with three different genotypes. There is no difference in hearing threshold between female and male knockout mice, therefore, both female and male mice were included in all experiments.

### Fluorescence *in situ* hybridization (FISH)

Frozen mouse cochlear sections were prepared in a routine fashion. Briefly, mouse cochlear tissue was fixed with 4% paraformaldehyde immediately after collection. The cochlea were decalcified with 10% EDTA, gradient dehydrated in 10 and 20% sucrose solutions, and then cryo-protected with 30% sucrose solution, embedded and frozen in OCT, and finally cut into 6-μm thick sections. Each slide was fixed with 4% paraformaldehyde for 10 min and washed with 0.01 M PBS three times for 5 min each. After incubation with 20 μg/ml proteinase K (10 min, 37°C) and prehybridization (1 h, 37°C), FISH was performed using a specific miR-29a probe (002112, Thermo Fisher Scientific, Shanghai, China), labeled with FAM (488) fluorochromes (excitation wavelength: 488 nm; emission wavelength: 515–555 nm). The tissue slices were also stained with 4′,6-diamidino-2-phenylindole (DAPI) (G1012, Servicebio, Wuhan, China) (excitation wavelength: 330–380 nm; emission wavelength: 420 nm).

### Auditory brainstem response (ABR)

Mice were anesthetized with an intraperitoneal (IP) injection of ketamine (100 mg/kg) and xylazine (10 mg/kg) and ABR was recorded monthly in a soundproof chamber. The signals of click and tone-burst stimuli (8, 16, and 32 kHz) were presented and channeled into the mouse’s ear canals. Amplified brainstem responses were recorded by Smart EP (Intelligent Hearing Systems, USA). The active electrode of three subdermal electrode needles was inserted at the vertex, the reference electrode was inserted under the test ear, and the ground electrode was inserted under the contralateral ear as previously described (Zheng et al., 1999; Wang et al., 2017; Zhao et al., 2020). Stimulus intensities were elicited in 10 dB descending steps, which were followed by a 5 dB increasing step until no response was detected. The ABR threshold was defined as the minimum sound intensity sufficient to elicit a reproducible wave II.

### Histological examination

Four-month-old *miR-29a*^+/+^ and *miR-29a^–/–^* mice were sacrificed. Paraffin-embedded mouse cochlear sections with a 5-μm thickness were prepared as described previously (Zhao et al., 2020). The tissue sections were stained with Hematoxylin and Eosin (H&E). Hair cells, stria vascularis, and spiral ganglion cells were observed under an optical microscope (Leica DMI4000 B, Germany). SGN counts were performed as previously described (Semaan et al., 2013; Fu et al., 2022). The width of SV and the area of the spiral ganglion region were measured with the aid of NIH ImageJ 1.53t software.

### Immunofluorescence of cochlear hair cells

After the collected cochlea was fixed and decalcified, it was microdissected and divided into three sections: basal turn, middle turn and apical turn. Tissues were permeabilized with 0.5% Triton X-100 for 15 min at room temperature and blocked with 5% goat serum for 1 h. The samples were first stained for hair cells with rabbit anti-Myosin VIIA (anti-MYO7A, Proteus BioSciences, #25- 6790, 1:150) at 4 C overnight. After the samples were washed three times with 0.01 M PBS, goat anti-rabbit IgG (H + L) secondary antibody (Alexa Fluor 594, Life Technologies, A11012, 1:500) was used together with phalloidin (Alexa 488, Invitrogen, A12379, 1:500) for 1 h at room temperature. Samples were washed another three times, and finally stained with DAPI (Invitrogen) for cell nuclei. Images were captured by a Zeiss LSM 880 laser scanning confocal microscope (Germany) equipped with a 40 × /0.95 N.A objective lens. Hair cell loss was assessed by the absence of MYO7A immunoreactivity. In the quantitative analysis of hair cell numbers, hair cells were counted over a length of 100 μm of the cochlea in the apical, middle, and basal turns, respectively. Three mice were examined per group.

### Electron microscopy

For scanning electron microscopy (SEM), the cochleas were fixed with 2.5% glutaraldehyde as previously described (Zhao et al., 2021). After they were rinsed in 0.01 M PBS, the cochleas were carefully microdissected to remove from the bone, spiral ligament, and Reissner’s membrane. Afterward, tissues were post-fixed in 1% osmium tetroxide (OsO_4_) for 1.5 h. Then specimens were rinsed again and dehydrated in graded ethanol of increasing concentration (30–100%). The BM was dried with CO_2_ at a critical point and coated with gold palladium. Finally, the specimens were viewed under a high-resolution scanning electron microscope (Hitachi S-4800, Japan).

The basement membrane samples used for transmission electron microscopy (TEM) were peeled from the cochlea. Then, they were fixed with glutaraldehyde, embedded in epoxy resin, and stained with uranyl acetate and lead citrate. TEM images were recorded by JEOL-1200E transmission electron microscope (Tokyo, Japan). For determination of the BM thickness, TEM images were analyzed using NIH ImageJ 1.53t software.

### RNA-seq analysis

Total RNA was extracted from the cochlea of 2-month-old miR-29a WT and miR-29a KO mice using TRIzol reagent. RNA quality control, library preparation, and sequencing were performed by Novogene (Tianjin, China). RNA-seq was performed on the Illumina platform (Novogene, China). Paired-end clean reads were mapped to the mouse reference genome using Hisat2 (v2.0.4). Differential expression analysis of two groups was identified using the DESeq2 R package (v1.30.0). Genes with a *P*-value < 0.05 and fold-change > 0 were considered as differentially expressed.

### Dual-luciferase reporter assay

Bioinformatics software TargetScan (release 7.1) and RegRNA (2.0) were used to predict the targeting relationship between miR-29a and ECM component genes based on the binding sites of miR-29a and the 3’-UTR of these genes (Supplementary Table 1).

The 3′-UTR of the mouse candidate target gene containing the miR-29a binding site (synthesized by Shanghai GeneChem) was inserted into psiCHECK-2 (Clontech) downstream of the luciferase gene using *Xho*I/*Not*I. The mutant 3′-UTR (deletion of the predicted binding site ACCACGA) of mouse miR-29a (synthesized by Shanghai GeneChem) was inserted into psiCHECK-2 downstream of the luciferase gene using *Xho*I/*Not*I. WT or mutant psiCHECK-2-targets with 3′-UTR binding sites and miR-29a mimic (synthesized by Shanghai GeneChem) were co-transfected into HEK 293T cells in 96-well culture dishes using Lipofectamine 2000. Forty-eight hours after transfection, firefly, and Renilla luciferase activities were measured in a GLOMAX luminometer using the Dual-Luciferase Reporter Assay System (Promega, E910). Luciferase activities were determined by Omega Software v5.50 R4.

### Inhibitor assay

Inhibitors for miR-29a and negative control miRNA were designed and synthesized by GenePharma Company. The corresponding sequences are summarized: mmu-miR-29a-3p inhibitor: 5′-UAACCGAUUUCAGAUGGUGCUA-3′, negative control miRNA inhibitor: 5′-CAGUACUUUUGUGUAGUACAA-3′. In all, each group at a final concentration of 100 nmol was transfected into HEI-OC1 cells in 6-well culture dishes using a riboFECT™ CP Transfection Kit (RiboBio). Forty-eight hours after transfection, total cellular RNA was extracted.

### RNA extraction and qRT-PCR

For cochlea tissue collection, the whole cochlea was carefully dissected out of the temporal bone as previously described (Fang et al., 2019; Seicol et al., 2022). Total RNA was extracted from cochlear tissues or cultured cells using cold TRIzol reagent (Ambion, Austin, TX, USA). RNA was reverse transcribed to cDNA by using the PrimeScript™ RT reagent kit (TaKaRa, Kusatsu, Japan). Real-time PCR was performed on a QuantStudio three instrument (Thermo Fisher Scientific) using SYBR Green PCR Master Mix (Roche, Basel, Switzerland). All primer sequences used in this study are shown in Supplementary Table 2. The qRT-PCR results were obtained by QuantStudio™ Design and Analysis SE Software v1.5.0. The relative expression levels of each mRNA were calculated after normalizing to β-actin. Data were expressed as 2^–ΔCt^ values with ΔCT = Ct_target gene_-Ct_referencegene_.

### Western blotting analysis

Mice cochlea samples were prepared as reported previously (Fuentes-Santamaria et al., 2022). Briefly, after mice were euthanized, the whole cochlea was quickly dissected from the temporal bone. Total protein extracts were obtained by lysing cochlear tissues in 100 μl of RIPA buffer (Thermo Scientific, Waltham, MA, USA), including a protease and phosphatase inhibitor cocktail (Roche, Switzerland). Protein samples were separated on an 8% SDS-PAGE gel (Beyotime, Nanton, China) and electrotransferred to a PVDF membrane (Invitrogen, Waltham, MA, USA). After the membrane was blocked, immunoblotting was performed with the following antibodies: anti-COL4A1 (1:50, Novus, NBP1-26549), anti-COL4A2 (1:250, Absin, abs140629), anti-COL4A3 (1:500, a kind gift from Dr. Jeffrey H. Miner, Washington University), anti-COL4A4 (1:500, a kind gift from Dr. Jeffrey H. Miner, Washington University), anti-COL4A5 (1:500, Abcam, ab231957), anti-LAMB2 (1:150, Santa Cruz, sc-377379), anti-LAMC1 (1:500, Cell Signaling Technology, #92921), and anti-β-actin (1:1000, Cell Signaling Technology, #4970). HRP-conjugated secondary antibodies (Abcam) followed by ECL (Meilunbio, Dalian, China, MA0186) incubation allowed protein band detection. Protein bands were visualized using an image acquisition and analysis system (Bio-Rad, Hercules, CA, USA). ImageJ 1.52a was used to quantify the Western blotting results.

### Immunofluorescence staining

For immunofluorescence of cochlear tissue, 5-μm frozen sections were fixed with 2% paraformaldehyde for 20 min at room temperature. After incubation with 0.3% Triton for 30 min, the frozen sections were blocked in 5% goat serum albumin for 2 h. Then, the samples were incubated with primary antibodies at 4°C overnight (Cheng et al., 2019). The primary antibodies used in this study were COL4A1 (IF: 1:10, Novus, NBP1-26549) and LAMB2 (IF:1:40, Santa Cruz, sc-377379). Alexa 647-conjugated secondary antibodies (1:400, Absin Bioscience) and Alexa 488-conjugated secondary antibodies (1:500, Life Technologies, Carlsbad, CA, USA) were used for 1 h. Finally, tissue sections were counterstained with DAPI for 5 min. Fluorescence images were obtained using a Zeiss laser scanning confocal microscope (Zeiss, LSM880, Jena, Germany).

### Statistical analysis

All data are shown as the mean ± SD. The significance of differences in ABR thresholds between groups was tested using a two-way analysis of variance (ANOVA) followed by Tukey’s multiple group matching test. For other experimental data between two groups, independent *t*-tests or paired *t*-tests were used. *P* < 0.05 was defined as statistically significant. All statistical analyses were performed in IBM SPSS Statistics 26.

## Results

### Genotyping of miR-29a KO and detection of miR-29a expression in mice

To confirm the expression of miR-29a, we genotyped all mice by polymerase chain reaction (PCR) using genomic DNA from tail clips. As shown in Supplementary Figure 1, wild-type *miR-29a*^+/+^ mice showed a single band of 480 bp, homozygous *miR-29a^–/–^* mice showed a band of 717 bp, and heterozygous *miR-29a*^+/–^ mice showed both bands (717 and 480 bp).

**FIGURE 1:**
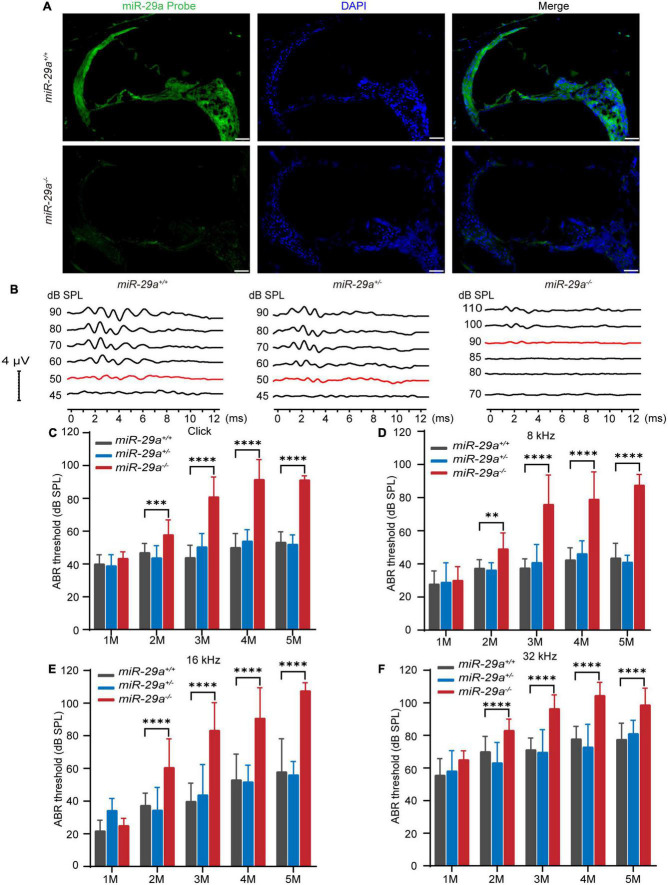
Expression of miR-29a in mouse cochlea and ABR in the miR-29a mouse strains. **(A)** Fluorescence *in situ* hybridization of frozen sections from mice. The cochlea labeled with a miR-29a probe for miR-29a (green) and DAPI for nuclei (blue) are shown in the images. *miR-29a*^+/+^ mice are shown in the upper panels, and *miR-29a^–/–^* mice are shown in the lower panels. Scale bar = 50 μm. Images are representative of *n* = 3 biological replicates per group. **(B)** Comparison of the representative ABR waveform of 4-month-old *miR-29a*^+/+^, *miR-29a*^+/–^, and *miR-29a^–/–^* mice were elicited by click. The changes in the mean ABR thresholds of *miR-29a^–/–^* mice at 1, 2, 3, 4, and 5 months of age indicate that *miR-29a^–/–^* mice have more severe hearing loss than *miR-29a*^+/+^ mice at all stimuli: click **(C)**, 8 kHz **(D)**, 16 kHz **(E)**, and 32 kHz **(F)**. Numbers of *miR-29a*^+/+^ mice tested in 1 (*n* = 9), 2 (*n* = 13), 3 (*n* = 22), 4 (*n* = 29), and 5 (*n* = 14) months old mice. Numbers of *miR-29a*^+/–^ mice tested in 1 (*n* = 13), 2 (*n* = 8), 3 (*n* = 12), 4 (*n* = 9), and 5 (*n* = 5) months old mice. Numbers of *miR-29a*^–/–^ mice tested in 1 (*n* = 6), 2 (*n* = 20), 3 (*n* = 17), 4 (*n* = 20), and 5 (*n* = 4) months old mice. All data are shown as the mean ± SD, two-way ANOVA followed by Tukey’s multiple comparisons test. Asterisks indicate significant of Tukey’s multiple comparisons tests (**P < 0.01, ***P < 0.001, and ****P < 0.0001).

The expression of miR-29a in the cochlea was detected by *in situ* hybridization. As shown in Figure 1A, miR-29 was expressed in most parts of the cochlea, including the organ of Corti, BM, stria vascularis, limbus spiralis, and spiral ganglion, whereas there was only background staining in the miR-29a KO mice.

### MiR-29a-deficiency causes progressive hearing loss

To determine the hearing function of miR-29a mutant strains, we measured their ABRs. We assessed ABR thresholds with click and tone bursts (8, 16, and 32 kHz) in miR-29a mutant strains aged 1 to 5 months (Figures 1B–F).

We compared the mean ABR threshold of *miR-29a*^–/–^ mice, wild-type *miR-29a*^+/+^ mice and heterozygous *miR-29a*^+/–^ mice from 1 to 5 months of age for click, 8, 16, and 32 kHz. A two-way ANOVA was used to assess the effect of age and genotype on the ABR threshold for each frequency. For click, the main effect of age was also significant [*F*_(4, 53)_ = 45.235, *P* < 0.0001], and the main effect of genotype was significant [*F*_(2, 55)_ = 145.536, *P* < 0.0001]. For 8 kHz, there were significant effects of age and genotype on the ABR threshold [*F*_(4, 53)_ = 37.039, *P* < 0.0001; *F*_(2, 55)_ = 100.828, *P* < 0.0001]. For 16 kHz, there were significant effects of age and genotype on the ABR threshold [*F*_(4, 53)_ = 39.993, *P* < 0.0001; *F*_(2, 55)_ = 69.268, *P* < 0.0001]. For 32 kHz, there were significant effects of age and genotype on the ABR threshold [*F*_(4, 53)_ = 36.600, *P* < 0.0001; *F*_(2, 55)_ = 68.782, *P* < 0.0001]. Tukey’s multiple comparison test results showed significant differences were observed between *miR-29a*^+/+^ mice and *miR-29a*^–/–^ mice for each stimulus from 2 to 5 months of age (*P* < 0.001), whereas the mean threshold from 1 to 5 months of age in the heterozygous *miR-29a*^+/–^ mice was not significantly different from that in wild-type *miR-29a*^+/+^ mice for click, 8, 16, and 32 kHz (all *P* > 0.05), as shown in Figures 1C–F. These results suggest that miR-29a-deficiency results in progressive, age-dependent hearing loss.

### Cochlear pathology of *miR-29a^–/–^* mice

**FIGURE 2:**
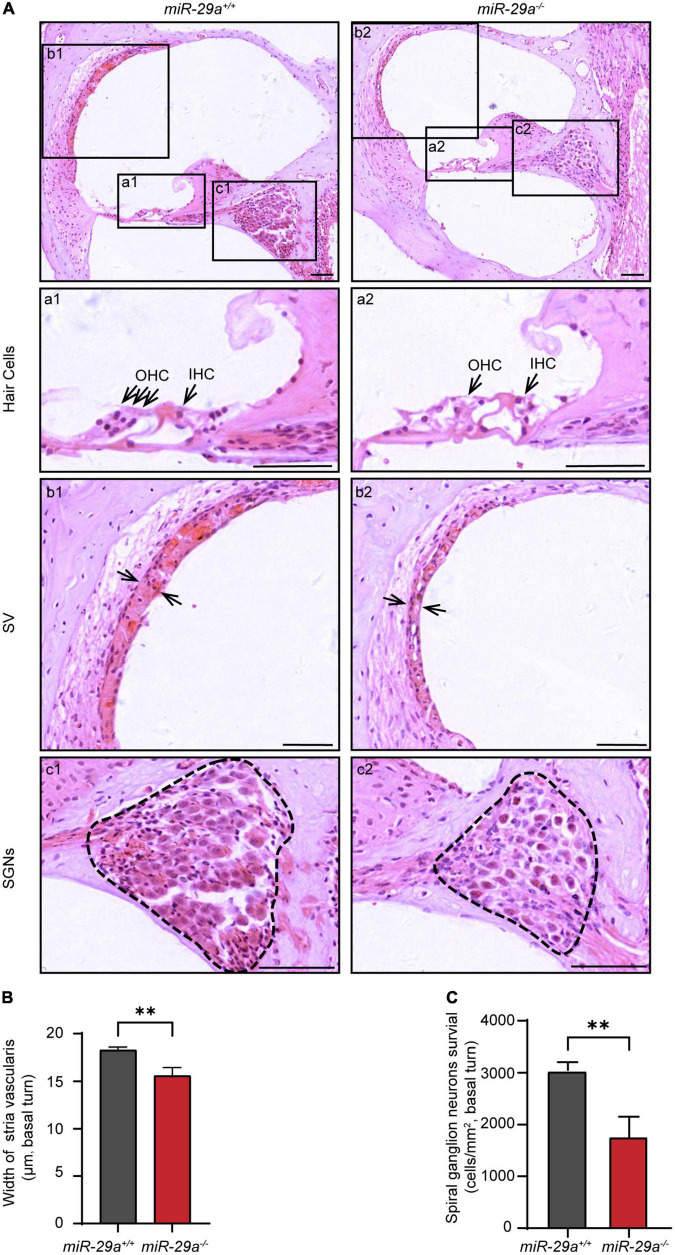
Morphological features in the cochlea of *miR-29a*^+/+^ and *miR-29a*^–/–^ mice at 4 months. **(A)** Loss of hair cells, spiral ganglion neurons (SGNs), and the thickness of stria vascularis (SV) in the basal turns of cochlea were observed by H&E staining. Scale bar = 50 μm. (a) Magnification of hair cells in the cochlea of *miR-29a*^+/+^ and *miR-29a*^–/–^ mice. Scale bar = 50 μm. (b) Magnification of SV in the cochlea of *miR-29a*^+/+^ and *miR-29a^–/–^* mice. Scale bar = 50 μm. (c) Magnification of SGNs, in the cochlea of *miR-29a*^+/+^ and *miR-29a^–/–^* mice. Scale bar = 50 μm. **(B)** The width of SV. The mean width of SV in the basal cochlear regions of *miR-29a^–/–^* mice was generally thinner than that of *miR-29a*^+/+^ mice at 4 months old [*t*_(4)_ = –5.096, *P* = 0.007]. **(C)** SGNs survival (i.e., SGNs density). The mean SGN density of the basal cochlear regions of *miR-29a^–/–^* mice was significantly lower than that of *miR-29a*^+/+^ mice at 4 months [*t*_(4)_ = –5.543, *P* = 0.005]. IHC, inner hair cell; OHC, outer hair cell; SV, stria vascularis; SGNs, spiral ganglion neurons. All data are shown as the mean ± SD, *n* = 3 biological replicates per group, the independent *t*-test was used to compare data between two groups (**P < 0.01).

We compared the histological morphology of the organs of Corti in the *miR-29a*^+/+^ and *miR-29a^–/–^* mice at 4 months of age. Hair cell loss was evident in the *miR-29a^–/–^* mice compared with the *miR-29a*^+/+^ mice (Figure 2A). The width of stria vascularis in the *miR-29a^–/–^* mice was thinner than that in the *miR-29a*^+/+^ mice (Figure 2B), and the difference was significant [independent *t*-test, *t*_(4)_ = −5.096, *P* = 0.007]. We also examined the loss of spiral ganglion neurons (SGNs) by measuring cell density (Figure 2C). The SGN density of the cochlear basal turns of the *miR-29a^–/–^* mice was significantly lower than that of the *miR-29a*^+/+^ mice at 4 months of age [independent *t*-test, *t*_(4)_ = 5.543, *P* = 0.005]. The abnormalities in these specific regions detected in the *miR-29a^–/–^* mice are consistent with the finding that these mice exhibited severe hearing loss at 4 months of age.

**FIGURE 3:**
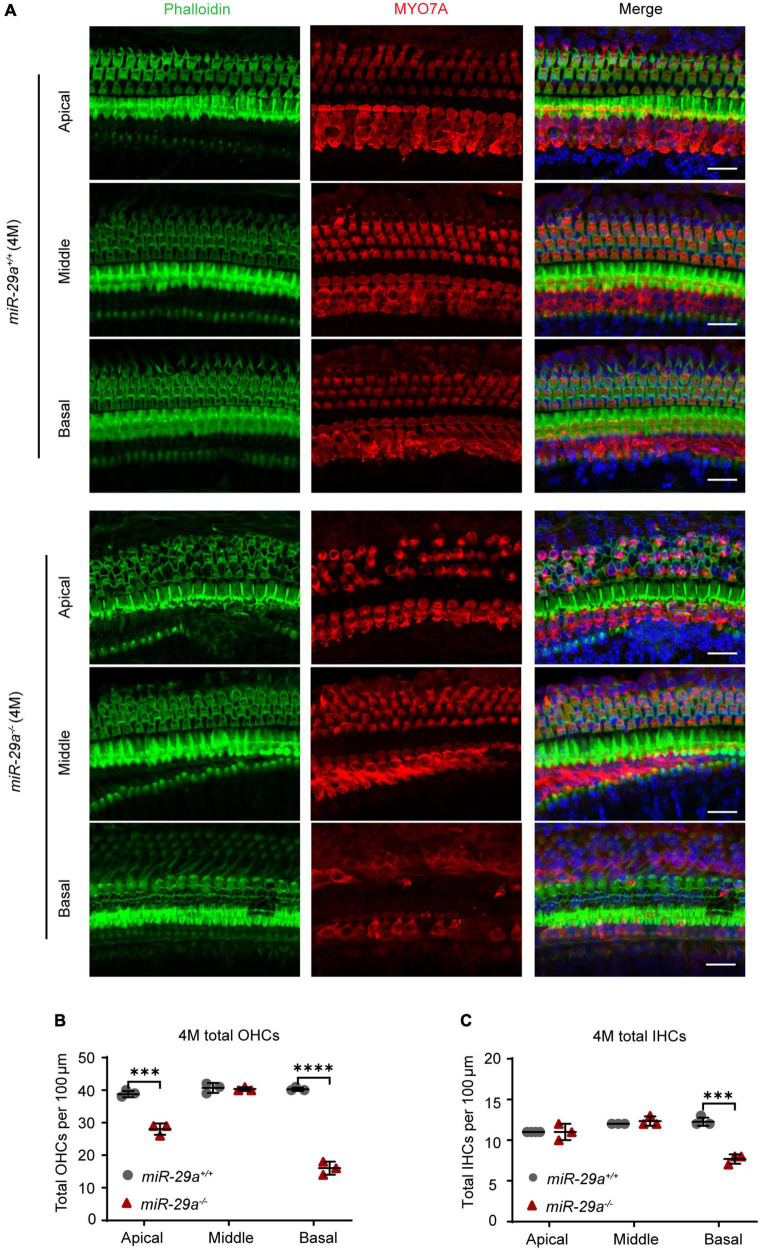
Hair cell loss in *miR-29a^–/–^* mice shown by confocal images. **(A)** *miR-29a*^+/+^ and *miR-29a^–/–^* mice display hair cell generation from the basal, middle, and apical regions of the cochlea at 4 months. Cochlear whole mounts labeled with phalloidin for filamentous actin (green), MYO7A for hair cells (red), and DAPI for nuclei (blue) are shown in confocal images. Scale bar = 20 μm. **(B)** Quantification of the total outer hair cell (OHCs) and inner hair cells (IHCs) **(C)** per 100 μm cochlear length at 4 months of age in *miR-29a^–/–^* and *miR-29a*^+/+^ mice [OHC: Apical, *t*_(4)_ = –9.526, *P* < 0.001; Middle, *t*_(4)_ = –0.354, *P* = 0.742; Basal, *t*_(4)_ = –20.247, *P* < 0.0001. IHC: Apical, *t*_(4)_ = 1.732, *P* = 0.158; Middle, *t*_(4)_ = 1.000, *P* = 0.374; Basal, *t*_(4)_ = –9.899, *P* < 0.001]. Data are shown as the mean ± SD, *n* = 3 biological replicates, ***P < 0.001 and ****P < 0.0001 between two groups.

To further investigate the hair cell pathology caused by the mutations, we observed hair cells in P5 and 4-month-old *miR-29a*^+/+^ and *miR-29a^–/–^* mice using surface preparation of cochlear epithelia. Hair cells were visualized by staining with MYO7A, phalloidin, and DAPI, and we evaluated hair cell survival based on MYO7A immunoreactivity. Confocal images showed that hair cells were intact in all turns of 5-day-old WT and KO mice (Supplementary Figure 2). At 4 months of age, hair cells of *miR-29a*^+/+^ mice were intact in all regions (Figure 3A), however, significant loss of OHCs was observed in the basal and apical turns of *miR-29a^–/–^* mice (Figure 3B), and significant loss of IHCs was observed in the basal turns (Figure 3C).

**FIGURE 4:**
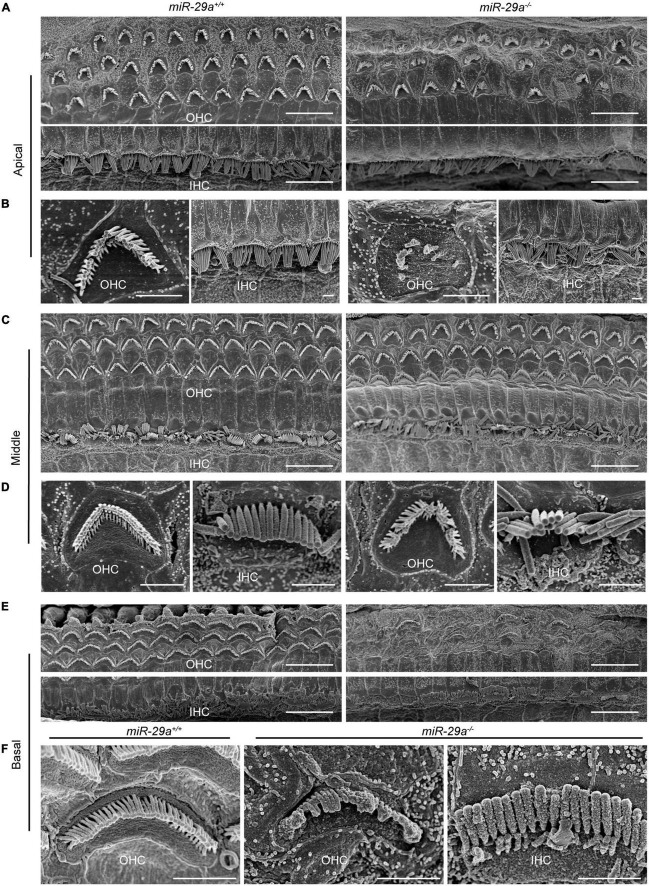
Representative images of the stereocilia of cochlear hair cells in *miR-29a*^+/+^ and *miR-29a^–/–^* mice shown by SEM. **(A–F)** *miR-29a*^+/+^ mice are on the left panels, and *miR-29a^–/–^* mice are on the right panels. **(A)** Global view of the apical region of the cochlea showing the presence of IHCs and OHCs in mice at 4 months of age. Scale bar = 10 μm. **(B)** Magnification images of OHCs and IHCs stereocilia in the cochlear apical region of *miR-29a*^+/+^ and *miR-29a^–/–^* mice. Scale bar = 2 μm. **(C)** Global view of the middle region of the cochlea showing the presence of IHCs and OHCs in mice at 4 months of age. Scale bar = 10 μm. **(D)** Magnification images of OHC and IHC stereocilia in the cochlear middle region of mice. Scale bar = 2 μm. **(E)** Global view of the basal region of the cochlea showing the presence of IHCs and OHCs in mice at 4 months of age. Scale bar = 10 μm. **(F)** Magnification images of OHC and IHC stereocilia in the cochlear basal region of mice. Scale bar = 2 μm. These images are representative images of five independent animals.

We also assessed the structural integrity of cochlear stereocilia by SEM. The control *miR-29a*^+/+^ mice showed the normal morphology of stereociliary bundles at 4 months of age, which were neatly arranged on the top of hair cells, and no hair cells were lost. In contrast, the *miR-29a^–/–^* mice displayed stereocilia fusion, degeneration and loss of hair cells in the basal and apical regions, except in the middle regions where hair cells remained intact (Figures 4C, D). As shown in Figures 4A, E, the stereocilia of the surviving OHCs lost their characteristic W-shaped and well-organized staircase pattern. Stereocilia fusion and degeneration were evident in many hair cells of *miR-29a^–/–^* mice (Figures 4B, F). These results suggest that homozygous deletion of miR-29a resulted in stereocilia fusion and missing hair cells.

### MiR-29a regulates the expression of major ECM component genes

**FIGURE 5:**
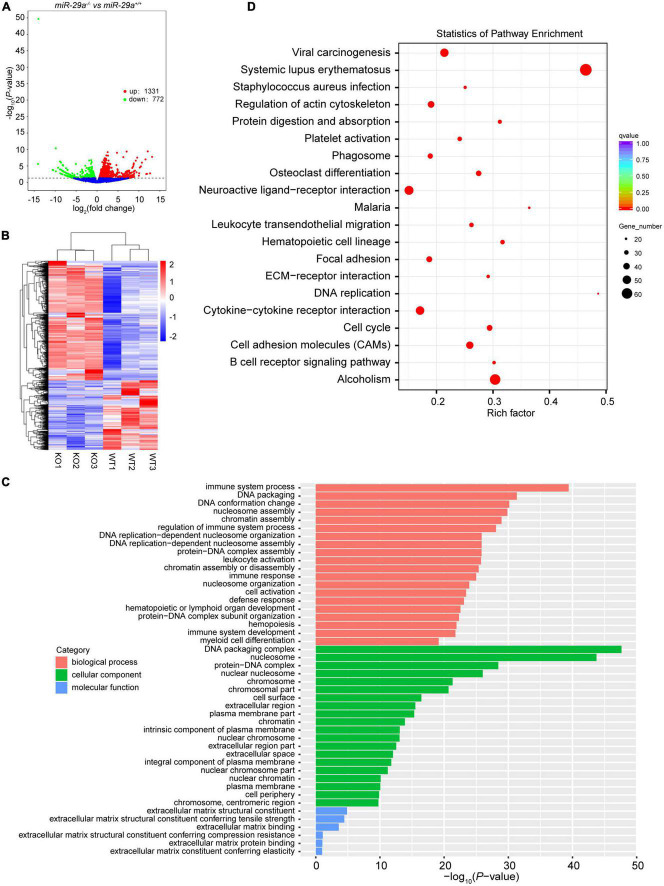
Transcriptome sequencing results of cochlea in *miR-29a*^+/+^ and *miR-29a^–/–^* mice. **(A)** Volcano plot of differentially expressed genes (miR-29a KO vs. WT). The *X*-axis is the log_2_Fold change value, and the *Y*-axis is the *P*-value. Genes with a *P*-value < 0.05 and | log_2_fold change| > 0 were considered as differentially expressed (*n* = 3 per group). **(B)** The clustering heat map of the differentially expressed mRNAs. **(C)** GO enrichment analysis of the differentially expressed genes. The *X*-axis is the *P*-value and the *Y*-axis is the GO term. **(D)** KEGG enrichment analysis was performed between miR-29a KO vs. WT. The top 20 significant pathways with adjusted *P*-values < 0.05 are shown.

To gain insight into what might explain the phenotype of miR-29a-deficiency in hearing loss, we performed RNA-seq on the cochlea of miR-29a KO and miR-29a WT mice. Compared with the WT group, the difference in mRNAs expression in KO group was statistically significant. There were a total of 2,103 differentially expressed mRNAs, of which 1,331 mRNAs were up-regulated and 772 mRNAs were down-regulated (Figures 5A, B). With *P* < 0.05 as the screening threshold, gene ontology (GO) enrichment analysis showed that differentially expressed mRNAs were highly correlated with terms related to ECM in both cell component and molecular function (Figure 5C). As shown in Figure 5D, with adjusted *P*-values < 0.05 as the screening threshold, the key signaling pathways enriched by Kyoto Encyclopedia of Genes and Genomes (KEGG) analysis also contain ECM-related pathways.

**FIGURE 6:**
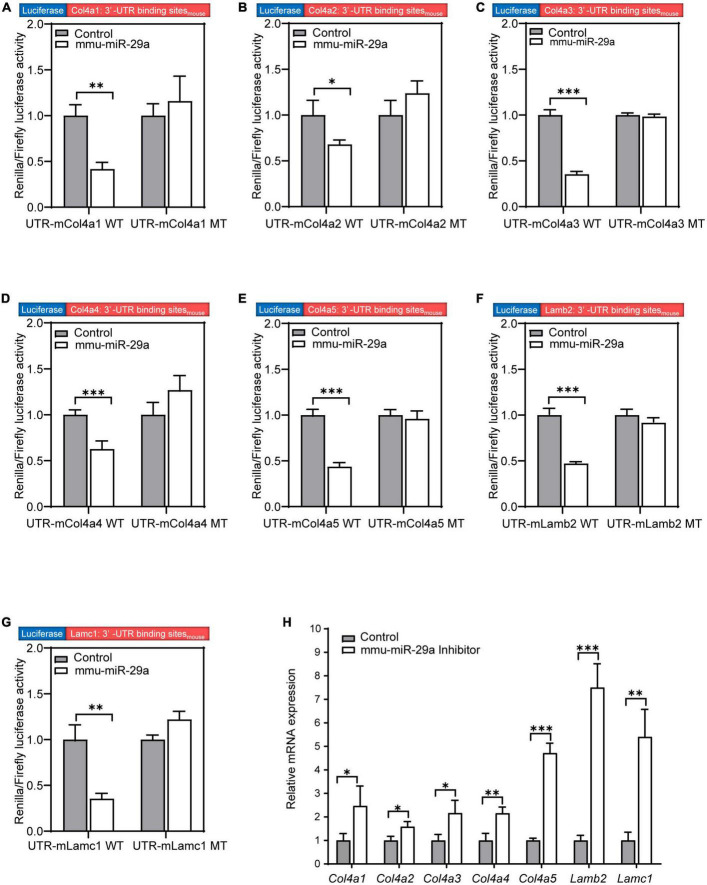
MiR-29a regulation of target gene expression in cell lines. **(A–G)** The Renilla/firefly luciferase activity measured in HEK293T cells of the wild-type (WT) psiCHECK2-Col4a1:3′-UTR binding site_mouse_ and mutant psiCHECK2-Col4a1:3′-UTR binding site_mouse_ cotransfected without (control) or with mmu-miR-29a mimics **(A)**. **(B)** WT or mutant psiCHECK2-Col4a2:3′-UTR binding site_mouse_ and without or with mmu-miR-29a mimics. **(C)** WT or mutant psiCHECK2-Col4a3:3′-UTR binding site_mouse_ and without or with mmu-miR-29a mimics. **(D)** WT or mutant psiCHECK2-Col4a4:3′-UTR binding site_mouse_ and without or with mmu-miR-29a mimics. **(E)** WT or mutant psiCHECK2-Col4a5:3′-UTR binding site_mouse_ and without or with mmu-miR-29a mimics. **(F)** WT or mutant psiCHECK2-Lamb2:3′-UTR binding site_mouse_ and without or with mmu-miR-29a mimics. **(G)** WT or mutant psiCHECK2-Lamc1:3′-UTR binding site_mouse_ and without or with mmu-miR-29a mimics. **(H)** Relative mRNA expression levels in HEI-OC1 cells transfected with mmu-miR-29a inhibitors. All data are shown as the mean ± SD, *n* = 4 biological replicates, statistical analysis was performed by paired *t*-test **(A–G)** and independent *t*-test **(H)**, where *P* < 0.05 was considered significant (*P < 0.05, **P < 0.01, and ***P < 0.001).

We searched the target gene prediction website^1^ for predicted target genes of miR-29a and found that the major ECM component genes *Col4a1*, *Col4a2*, *Col4a3*, *Col4a4*, *Col4a5*, *Lamb2*, and *Lamc1* are all target gene candidates. To verify whether the expression of these seven targets was directly regulated by miR-29a, we used dual-luciferase reporter assays based on luciferase constructs harboring WT or mutated (MT) miR-29a miRNA response element (MRE) sequences in the 3′-UTR. HEK293T cells were transfected with Col4a1-3′-UTR-WT and Col4a1-3′-UTR-MT after control or mmu-miR-29a mimic treatment. The statistical analysis indicated that the luciferase activity decreased in the Col4a1-3′-UTR-WT + miR-29a mimic group compared with the control Col4a1-3′-UTR-WT group [paired *t*-test, *t*_(3)_ = −8.312, *P* = 0.004], while the luciferase activity exhibited no significant difference between the control Col4a1-3′-UTR-MT and Col4a1-3′-UTR-MT + miR-29a mimic groups [paired *t*-test, *t*_(3)_ = 0.875, *P* = 0.446] (Figure 6A). All six other target genes were also verified in the same way, and the significant differences were consistent with the Col4a1 results. Overexpression of mmu-miR-29a significantly inhibited the expression of the reporter gene containing the WT 3′-UTR of the target gene [paired *t*-test, UTR-mCol4a2 WT, *t*_(3)_ = −4.239, *P* = 0.024; UTR-mCol4a3 WT, *t*_(3)_ = −20.931, *P* < 0.001; UTR-mCol4a4 WT, *t*_(3)_ = −17.998, *P* < 0.001; UTR-mCol4a5 WT, *t*_(3)_ = −13.117, *P* < 0.001; UTR-mLamb2 WT, *t*_(3)_ = −17.079, *P* < 0.001; UTR-mLamc1 WT, *t*_(3)_ = −6.581, *P* = 0.007], whereas it had no significant effect on the reporter gene containing mutated mmu-miR-29a binding sites [paired *t*-test, UTR-mCol4a2 MT, *t*_(3)_ = 1.804, *P* = 0.169; UTR-mCol4a3 MT, *t*_(3)_ = −0.666, *P* = 0.553; UTR-mCol4a4 MT, *t*_(3)_ = 1.933, *P* = 0.149; UTR-mCol4a5 MT, *t*_(3)_ = −0.714, *P* = 0.527; UTR-mLamb2 MT, *t*_(3)_ = −1.785, *P* = 0.172; UTR-mLamc1 MT, *t*_(3)_ = 3.735, *P* = 0.033], as shown in Figures 6B–G.

To further verify the results, we used qRT-PCR to measure the mRNA expression levels of these target genes after the transfection of miR-29a inhibitors into HEI-OC1 cells. Compared with those of the control group, the mRNA expression levels of these seven targets in HEI-OC1 cells were significantly increased after miR-29a inhibitor treatment [Figure 6H, independent *t*-test, Col4a1, *t*_(4)_ = 2.834, *P* = 0.047; Col4a2, *t*_(4)_ = 3.633, *P* = 0.022; Col4a3, *t*_(4)_ = 3.632, *P* = 0.028; Col4a4, *t*_(4)_ = 5.000, *P* = 0.007; Col4a5, *t*_(4)_ = 14.989, *P* < 0.001; Lamb2, *t*(_4_) = 10.909, *P* < 0.001; Lamc1, *t*_(4)_ = 6.227, *P* = 0.003, all *P* < 0.05]. These results suggest that the expression of these seven genes is directly regulated by miR-29a.

### Increased ECM gene expression in the cochlea of miR-29a knockout mice

**FIGURE 7:**
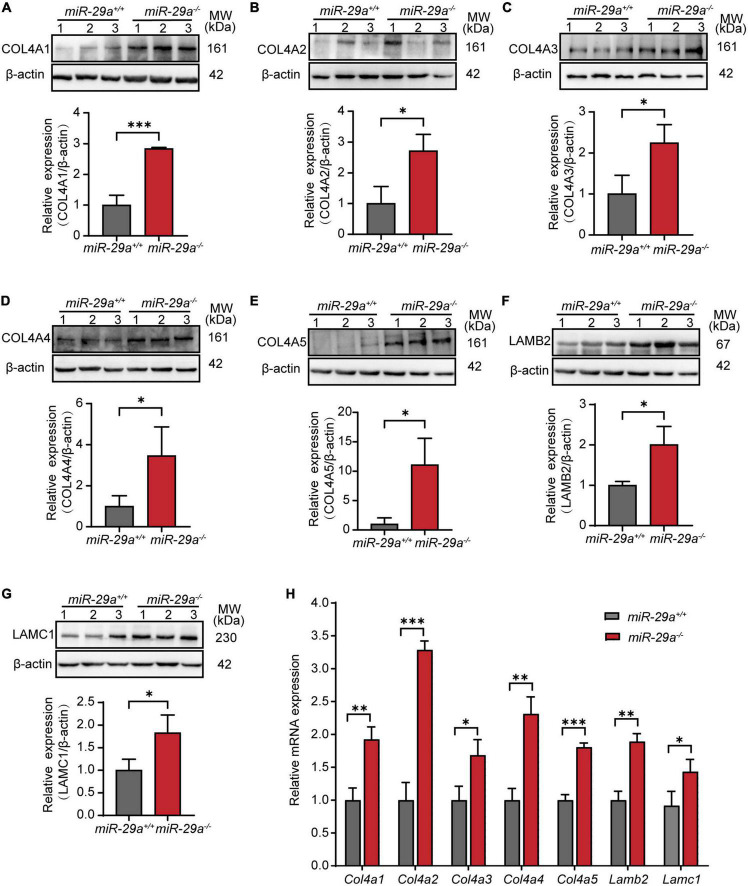
MiR-29a regulation of target gene expression in cochlea from *miR-29a*^+/+^ and *miR-29a^–/–^* mice. **(A–G)** With β-actin as a control, COL4A1, COL4A2, COL4A3, COL4A4, COL4A5, LAMB2, and LAMC1 protein levels in cochlea were analyzed by Western blot. The independent *t*-test was used to compare data between two groups [COL4A1, *t*_(4)_ = 9.920, *P* < 0.001; COL4A2, *t*_(4)_ = 3.847, *P* = 0.018; COL4A3, *t*_(4)_ = 3.389, *P* = 0.028; COL4A4, *t*_(4)_ = 2.842, *P* = 0.047; COL4A5, *t*_(4)_ = 3.782, *P* = 0.019; LAMB2, *t*_(4)_ = 3.915, *P* = 0.017; LAMC1, *t*_(4)_ = 3.088, *P* = 0.037]. **(H)** The mRNA expression levels of target genes as measured by qRT-PCR [independent *t*-test, Col4a1, *t*_(4)_ = 6.050, *P* = 0.004; Col4a2, *t*_(4)_ = 13.145, *P* < 0.001; Col4a3, *t*_(4)_ = 3.735, *P* = 0.02; Col4a4, *t*_(4)_ = 7.238, *P* = 0.002; Col4a5, *t*_(4)_ = 13.141, *P* < 0.001; Lamb2, *t*_(4)_ = 8.529, *P* = 0.001; Lamc1, *t*_(4)_ = 3.126, *P* = 0.035]. All data are shown as the mean ± SD, *n* = 3 biological replicates, *P < 0.05, **P < 0.01, and ***P < 0.001 between two groups.

To examine the expression of the above validated target genes in the miR-29a KO mice, we performed Western blot, qRT-PCR and immunofluorescence analyses in the *miR-29a*^+/+^ and *miR-29a^–/–^* mice. Western blot analysis showed that the protein expression levels of COL4A1 (Figure 7A), COL4A2 (Figure 7B), COL4A3 (Figure 7C), COL4A4 (Figure 7D), COL4A5 (Figure 7E), LAMB2 (Figure 7F), and LAMC1 (Figure 7G) were significantly increased in the *miR-29a^–/–^* mice compared with the *miR-29a*^+/+^ mice.

Moreover, in our study, the mRNA expression levels of *Col4a1*, *Col4a2*, *Col4a3*, *Col4a4*, *Col4a5*, *Lamb2*, and *Lamc1* were much higher in cochlea from the miR-29a*^–/–^* mice than in those from the control littermates (Figure 7H).

**FIGURE 8:**
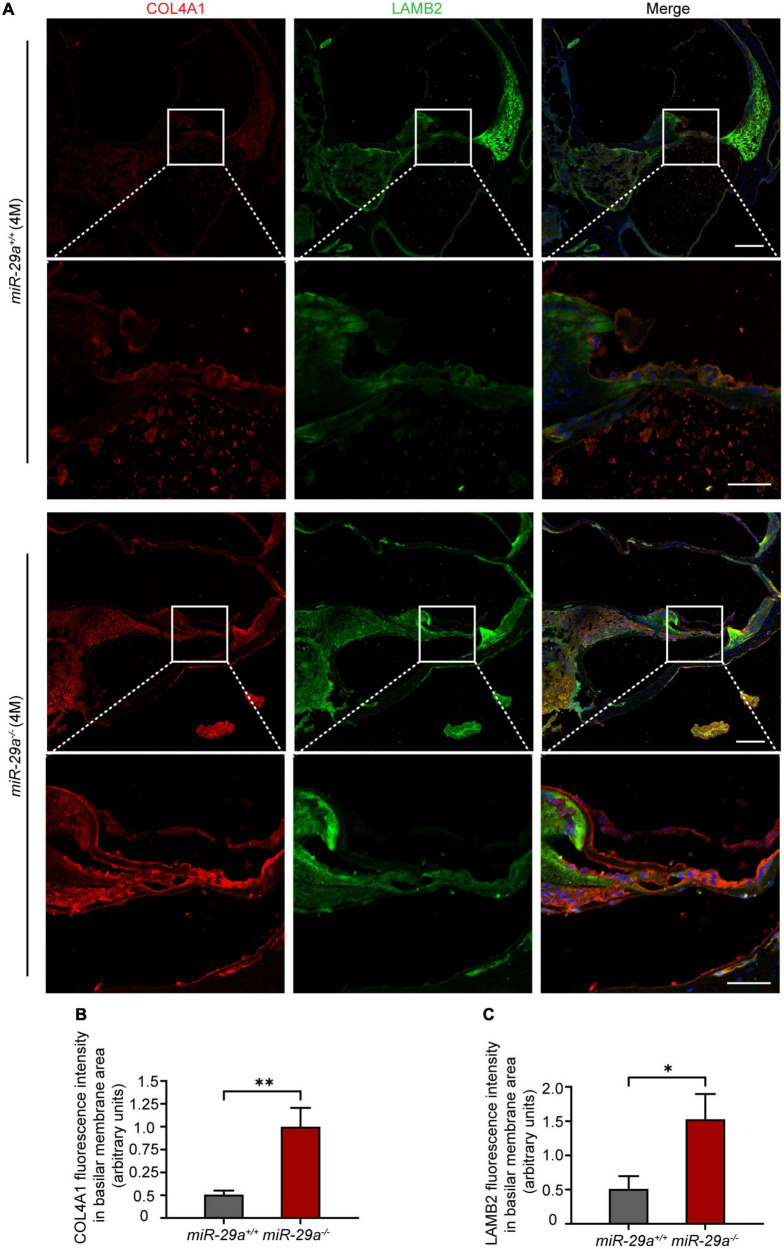
Specific expression of COL4A1 and LAMB2 in the basilar membrane (BM) area. **(A)** Double immunostaining for COL4A1 (red) and LAMB2 (green) in cochlear frozen sections from *miR-29a*^+/+^ and *miR-29a^–/–^* mice at 4 months. Nuclei were visualized by DAPI (blue). Scale bar = 100 μm for global images, scale bar = 50 μm for higher magnification. **(B,C)** Quantification of COLA1 and LAMB2 immunofluorescence in the BM area [independent *t*-test, COL4A1, *t*_(4)_ = 5.867, *P* = 0.004; Lamb2, *t*_(4)_ = 4.143, *P* = 0.014]. Data are shown as the mean ± SD, *n* = 3 biological replicates, *P < 0.05 and **P < 0.01 between two groups.

We selected Col4a1 and Lamb2 as representative genes to record immunoreactivity from cochlear frozen sections. Immunofluorescence staining of frozen sections revealed intense COL4A1 and LAMB2 staining on the BM (Figure 8A). The mean fluorescence intensities of COL4A1 (Figure 8B) and LAMB2 (Figure 8C) in the *miR-29a^–/–^* mice were both significantly higher than those in the *miR-29a*^+/+^ mice. These results demonstrated that loss of miR-29a significantly enhances the expression of these target genes on the BM. Collagen and laminin are major ECM components that collectively form the BM (Subramanian et al., 2018). This finding prompted us to speculate that homozygous deletion of miR-29a might lead to changes in the cochlear BM.

### MiR-29a deficiency leads to thickening of the BM

**FIGURE 9:**
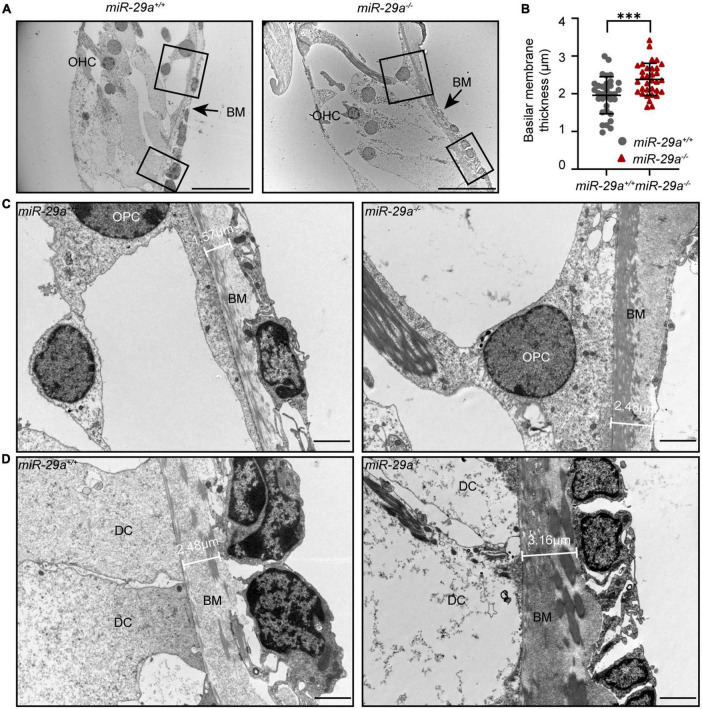
The thickness of the BM in the basal turn portrayed with TEM. **(A)** Radial section of the BM in the basal turn (high-frequency region) of cochlea from *miR-29a*^+/+^ and *miR-29a^–/–^* mice at 4 months. Scale bar = 20 μm. **(B)** Quantification of BM thickness [*n* = 3 independent animals and 10 images per group, independent *t*-test, *t*_(64)_ = 3.715, *P* = 0.0004, and ***P < 0.001]. **(C,D)** Framed area in panel **(A)** is shown at higher magnification. *miR-29a*^+/+^ mice are shown in the left panels, and *miR-29a^–/–^* mice are shown in the right panels. **(C)** BM thickness beneath outer pillar cells. Scale bar = 2 μm. **(D)** BM thickness beneath Deiters cells. Scale bar = 2 μm. BM, basilar membrane; OHC, outer hair cell; OPC, outer pillar cell; DC, Deiters’ cell.

To investigate whether there are changes in the structure of the BM, we examined the ultrastructure of cochlea from the *miR-29a*^+/+^ and *miR-29a^–/–^* mice by TEM. TEM showed pronounced thickening of the BM in the basal turn from the *miR-29a^–/–^* cochlea (Figures 9A, B). In addition, there were more collagen fibers in the BM beneath the outer pillar cells (Figure 9C) and Deiters’ cells (Figure 9D). In summary, mice with miR-29a deficiency showed alterations in thickness in the cochlear BM.

## Discussion

Hundreds of miRNAs were reported to be specifically expressed in the inner ear and can affect auditory function (Weston et al., 2006; Friedman et al., 2009; Elkan-Miller et al., 2011; Rudnicki and Avraham, 2012; Ushakov et al., 2013). However, the role of miR-29a in the cochlea is unknown and needs to be further studied and clarified. In this study, our data showed that miR-29a was expressed in the organ of Corti in the cochlea, and homozygous deletion of miR-29a in C57BL/6 mice caused progressive hearing loss and thickening of the cochlear BM.

Auditory brainstem response tests show that hearing loss in the miR-29a KO mice started with high frequencies and progressed to the middle and lower frequencies with age. This finding is consistent with the characteristics of ARHL (Bainbridge et al., 2011). ARHL occurs in most mammalian species and is closely associated with degenerative changes in auditory sensory cells, especially OHCs (Fu et al., 2021). Since ARHL is characterized by pronounced high-frequency hearing loss, it is generally accepted that the loss of sensory cells in the basal turn is the major cochlear pathology of aging (Keithley, 2020). However, Bredberg (1968) performed a detailed analysis of OHCs in 125 cochleae from 78 individuals, and his experiments documented OHC degeneration in both the basal turn and the apical turn of the cochlea. Another quantitative study of cochlear sensory cell loss in elderly individuals also confirmed that the degeneration of auditory sensory cells in humans, especially OHCs, begins in the basal turn and the apical turn (Wu et al., 2019). Consistent with these studies, we observed hair cells under a confocal laser scanning microscope and stereocilia bundles using a scanning electron microscope. We found that miR-29a-deficient mice showed more severe loss of OHCs and degeneration of stereocilia bundles in the basal and apical turns of the cochlea. Therefore, *miR-29a^–/–^* mice may serve as a potential animal model for the study of ARHL.

The mammalian inner ear is rich in basement membranes (White et al., 2022). There is a continuous layer of basement membrane in the cochlea that begins at the limbus, passes through the BM, and extends up to the spiral prominence (Cosgrove et al., 1998). Changes in basement membranes are thought to mediate the pathology of hearing impairment, including Alport syndrome and ARHL (Calzada et al., 2012; Meehan et al., 2016). Alport syndrome is an inherited disorder characterized by progressive glomerulonephritis with high-frequency hearing loss (Gratton et al., 2005). The pathogenesis of the inner ear in Alport syndrome was reported to be associated with thickening of the basement membrane (Cosgrove et al., 1998). Significant thickening of the basement membrane was observed in the inner ear of gerbils with an age-dependent decline in auditory function (Sakaguchi et al., 1997). Here, we found that the BM in the basal turns of the cochlea was significantly thickened in homozygous deletion miR-29a mice under TEM. Our data further indicate that thickening of the BM is closely related to hearing loss.

In addition to its well-recognized role as a structural support, the ECM is important for the homeostasis of adjacent cells in the body and is involved in many important functions (Birch, 2018). Basement membranes are highly specialized ECMs (Miner, 1999; Kruegel and Miosge, 2010; Mao et al., 2015; Wilson et al., 2020). Collagen IV and laminins are the core components of all basement membranes, including the BM (Cosgrove et al., 1996; Mak and Mei, 2017; Pozzi et al., 2017; Motta et al., 2019), and they are responsible for the strength and integrity of the basement membrane (Parkin et al., 2011). In this study, we validated several genes encoding collagen IV and laminins as direct targets of miR-29a-3p, including *Col4a1*, *Col4a2*, *Col4a3*, *Col4a4*, *Col4a5*, *Lamb2*, and *Lamc1*, by dual-luciferase reporter assays and qPCR. More importantly, the expression levels of these seven targets were significantly increased in the *miR-29a^–/–^* cochlea. Our *in situ* hybridization experiment showed that miR-29a was expressed in most parts of the cochlea, and the abnormalities we observed in the *miR-29a^–/–^* mice, including loss of hair cells, thinning stria vascularis and loss of SGNs, were likely due to the upregulation of these target genes. These results suggest that miR-29a plays an important role in maintaining the structural integrity of BM by regulating the synthesis of its core components collagen IV and laminins.

In summary, miR-29a-deficiency causes progressive hearing loss, which can be explained by the upregulated collagen IV and laminin leading to thickening of the BM, ultimately resulting in the loss of sensory hair cells. However, the exact molecular mechanisms by which thickening BMs lead to the loss of OHCs need further study. Overall, this study revealed a previously undescribed function and an important regulatory role for miR-29a in hearing loss. Our experiments also offer good evidence to suggest that miR-29a might be developed into a candidate therapeutic target for ARHL caused by BM changes.

## Data availability statement

The data presented in this study are deposited in the NCBI’s Gene Expression Omnibus (GEO) repository, accession number: GSE228284.

## Ethics statement

This animal study was reviewed and approved by the Institutional Animal Care and Use Committee of Binzhou Medical University.

## Author contributions

PM and SW performed the animal characterization and *in vitro* studies. YD, THZ, BL, and TZ performed the histologic analysis. ML and DX performed the mice genotyping. QZ, RG, and YG designed and supervised the study. PM analyzed the data and wrote the manuscript. All authors read and approved the final manuscript, and contributed to the study conception and design.

## Footnotes

1
http://www.targetscan.org

## References

[B1] Bainbridge K. E. Hoffman H. J. Cowie C. C. (2011). Risk factors for hearing impairment among U.S. adults with diabetes: National Health and Nutrition Examination Survey 1999-2004. *Diabetes Care* 34 1540–1545. 10.2337/dc10-2161 21593298 PMC3120175

[B2] Birch H. L. (2018). Extracellular matrix and ageing. *Subcell Biochem.* 90 169–190. 10.1007/978-981-13-2835-0_7 30779010

[B3] Boon R. A. Seeger T. Heydt S. Fischer A. Hergenreider E. Horrevoets A. (2011). MicroRNA-29 in aortic dilation: implications for aneurysm formation. *Circ. Res.* 109 1115–1119. 10.1161/CIRCRESAHA.111.255737 21903938

[B4] Bredberg G. (1968). Cellular pattern and nerve supply of the human organ of Corti. *Acta Otolaryngol.* 236:231. 4886545

[B5] Calzada A. P. Lopez I. A. Beltran Parrazal L. Ishiyama A. Ishiyama G. (2012). Cochlin expression in vestibular endorgans obtained from patients with Meniere’s disease. *Cell Tissue Res.* 350 373–384. 10.1007/s00441-012-1481-x 22992960 PMC4420027

[B6] Cheng C. Wang Y. Guo L. Lu X. Zhu W. Muhammad W. (2019). Age-related transcriptome changes in Sox2+ supporting cells in the mouse cochlea. *Stem Cell Res. Ther.* 10:365. 10.1186/s13287-019-1437-0 31791390 PMC6889721

[B7] Cosgrove D. Samuelson G. Meehan D. T. Miller C. McGee J. Walsh E. (1998). Ultrastructural, physiological, and molecular defects in the inner ear of a gene-knockout mouse model for autosomal Alport syndrome. *Hear. Res.* 121 84–98. 10.1016/s0378-5955(98)00069-0 9682811

[B8] Cosgrove D. Samuelson G. Pinnt J. (1996). Immunohistochemical localization of basement membrane collagens and associated proteins in the murine cochlea. *Hear. Res.* 97 54–65. 8844186

[B9] Dooley J. Lagou V. Garcia-Perez J. E. Himmelreich U. Liston A. (2017). miR-29a-deficiency does not modify the course of murine pancreatic acinar carcinoma. *Oncotarget* 8 26911–26917. 10.18632/oncotarget.15850 28460473 PMC5432306

[B10] Elkan-Miller T. Ulitsky I. Hertzano R. Rudnicki A. Dror A. Lenz D. (2011). Integration of transcriptomics, proteomics, and microRNA analyses reveals novel microRNA regulation of targets in the mammalian inner ear. *PLoS One* 6:e18195. 10.1371/journal.pone.0018195 21483685 PMC3071727

[B11] Fang Q. J. Wu F. Chai R. Sha S. (2019). Cochlear Surface Preparation in the Adult Mouse. *J. Vis. Exp*. 153:10.3791/60299. 10.3791/60299 31762458 PMC7217453

[B12] Friedman L. M. Dror A. A. Mor E. Tenne T. Toren G. Satoh T. (2009). MicroRNAs are essential for development and function of inner ear hair cells in vertebrates. *Proc. Natl. Acad. Sci. U. S. A.* 106 7915–7920. 10.1073/pnas.0812446106 19416898 PMC2683084

[B13] Fu X. An Y. Wang H. Li P. Lin J. Yuan J. (2021). Deficiency of Klc2 Induces Low-Frequency Sensorineural Hearing Loss in C57BL/6 J Mice and Human. *Mol. Neurobiol.* 58 4376–4391. 10.1007/s12035-021-02422-w 34014435

[B14] Fu X. Li P. Zhang L. Song Y. An Y. Zhang A. (2022). Activation of Rictor/mTORC2 signaling acts as a pivotal strategy to protect against sensorineural hearing loss. *Proc. Natl. Acad. Sci. U. S. A.* 119:e2107357119. 10.1073/pnas.2107357119 35238644 PMC8917383

[B15] Fuentes-Santamaria V. Alvarado J. C. Mellado S. Melgar-Rojas P. Gabaldón-Ull M. Cabanes-Sanchis J. (2022). Age-Related Inflammation and Oxidative Stress in the Cochlea Are Exacerbated by Long-Term, Short-Duration Noise Stimulation. *Front. Aging Neurosci.* 14:853320. 10.3389/fnagi.2022.853320 35450058 PMC9016828

[B16] Gratton M. A. Rao V. H. Meehan D. T. Askew C. Cosgrove D. (2005). Matrix metalloproteinase dysregulation in the stria vascularis of mice with Alport syndrome: implications for capillary basement membrane pathology. *Am. J. Pathol.* 166 1465–1474. 10.1016/S0002-9440(10)62363-2 15855646 PMC1606400

[B17] Guo L. Huang X. Liang P. Zhang P. Zhang M. Ren L. (2018). Role of XIST/miR-29a/LIN28A pathway in denatured dermis and human skin fibroblasts (HSFs) after thermal injury. *J. Cell Biochem.* 119 1463–1474. 10.1002/jcb.26307 28771809

[B18] Han Z. Zhang T. He Y. Li G. Li G. Jin X. (2018). Inhibition of prostaglandin E2 protects abdominal aortic aneurysm from expansion through regulating miR-29b-mediated fibrotic ECM expression. *Exp. Ther. Med.* 16 155–160. 10.3892/etm.2018.6160 29896234 PMC5995085

[B19] He Z. H. Li M. Fang Q. J. Liao F. Zou S. Wu X. (2021). FOXG1 promotes aging inner ear hair cell survival through activation of the autophagy pathway. *Autophagy* 17 4341–4362. 10.1080/15548627.2021.1916194 34006186 PMC8726647

[B20] He Z. H. Zou S. Y. Li M. Liao F. Wu X. Sun H. (2020). The nuclear transcription factor FoxG1 affects the sensitivity of mimetic aging hair cells to inflammation by regulating autophagy pathways. *Redox. Biol.* 28:101364. 10.1016/j.redox.2019.101364 31731101 PMC6920089

[B21] Horita M. Farquharson C. Stephen L. A. (2021). The role of miR-29 family in disease. *J. Cell Biochem.* 122 696–715. 10.1002/jcb.29896 33529442 PMC8603934

[B22] Hubmacher D. Apte S. S. (2013). The biology of the extracellular matrix: novel insights. *Curr. Opin. Rheumatol.* 25 65–70. 10.1097/BOR.0b013e32835b137b 23143224 PMC3560377

[B23] Jiang P. Ma X. Han S. Ma L. Ai J. Wu L. (2022). Characterization of the microRNA transcriptomes and proteomics of cochlear tissue-derived small extracellular vesicles from mice of different ages after birth. *Cell Mol. Life Sci.* 79:154. 10.1007/s00018-022-04164-x 35218422 PMC11072265

[B24] Keithley E. M. (2020). Pathology and mechanisms of cochlear aging. *J. Neurosci. Res.* 98 1674–1684. 10.1002/jnr.24439 31066107 PMC7496655

[B25] Kim T. S. Chung J. W. (2019). Associations of Dietary Riboflavin, Niacin, and Retinol with Age-related Hearing Loss: An Analysis of Korean National Health and Nutrition Examination Survey Data. *Nutrients* 11:896. 10.3390/nu11040896 31010085 PMC6520829

[B26] Kruegel J. Miosge N. (2010). Basement membrane components are key players in specialized extracellular matrices. *Cell Mol. Life Sci.* 67 2879–2895. 10.1007/s00018-010-0367-x 20428923 PMC2921489

[B27] Lewis M. A. Quint E. Glazier A. M. Fuchs H. De Angelis M. Langford C. (2009). An ENU-induced mutation of miR-96 associated with progressive hearing loss in mice. *Nat. Genet.* 41 614–618. 10.1038/ng.369 19363478 PMC2705913

[B28] Liu X. Z. Yan D. (2007). Ageing and hearing loss. *J. Pathol.* 211 188–197. 10.1002/path.2102 17200945

[B29] Lyu A. R. Kim T. H. Park S. J. Shin S. Jeong S. Yu Y. (2020). Mitochondrial Damage and Necroptosis in Aging Cochlea. *Int. J. Mol. Sci.* 21:2505. 10.3390/ijms21072505 32260310 PMC7177801

[B30] Ma R. Wang M. Gao S. Zhu L. Yu L. Hu D. (2020). miR-29a Promotes the Neurite Outgrowth of Rat Neural Stem Cells by Targeting Extracellular Matrix to Repair Brain Injury. *Stem Cells Dev.* 29 599–614. 10.1089/scd.2019.0174 31885334

[B31] Mak K. M. Mei R. (2017). Basement Membrane Type IV Collagen and Laminin: An Overview of Their Biology and Value as Fibrosis Biomarkers of Liver Disease. *Anat. Rec.* 300 1371–1390. 10.1002/ar.23567 28187500

[B32] Mao M. Alavi M. V. Labelle-Dumais C. Gould D. (2015). Type IV Collagens and Basement Membrane Diseases: Cell Biology and Pathogenic Mechanisms. *Curr. Top. Membr.* 76 61–116. 10.1016/bs.ctm.2015.09.002 26610912

[B33] Meehan D. T. Delimont D. Dufek B. Zallocchi M. Phillips G. Gratton M. (2016). Endothelin-1 mediated induction of extracellular matrix genes in strial marginal cells underlies strial pathology in Alport mice. *Hear. Res.* 341 100–108. 10.1016/j.heares.2016.08.003 27553900 PMC5086449

[B34] Mencia A. Modamio-Hoybjor S. Redshaw N. Morín M. Mayo-Merino F. Olavarrieta L. (2009). Mutations in the seed region of human miR-96 are responsible for nonsyndromic progressive hearing loss. *Nat. Genet.* 41 609–613. 10.1038/ng.355 19363479

[B35] Miner J. H. (1999). Renal basement membrane components. *Kidney Int.* 56 2016–2024. 10.1046/j.1523-1755.1999.00785.x 10594777

[B36] Motta C. M. Endres K. J. Wesdemiotis C. Willits R. Becker M. (2019). Enhancing Schwann cell migration using concentration gradients of laminin-derived peptides. *Biomaterials* 218:119335. 10.1016/j.biomaterials.2019.119335 31302351 PMC6868524

[B37] Noben-Trauth K. Zheng Q. YJohnson K. R. (2003). Association of cadherin 23 with polygenic inheritance and genetic modification of sensorineural hearing loss. *Nat. Genet.* 35 21–23. 10.1038/ng1226 12910270 PMC2864026

[B38] Noguchi Y. Kurima K. Makishima T. de Angelis M. Fuchs H. Frolenkov G. (2006). Multiple quantitative trait loci modify cochlear hair cell degeneration in the Beethoven (Tmc1Bth) mouse model of progressive hearing loss DFNA36. *Genetics* 173 2111–2119. 10.1534/genetics.106.057372 16648588 PMC1569729

[B39] Papadopoulou A. S. Dooley J. Linterman M. A. Pierson W. Ucar O. Kyewski B. (2011). The thymic epithelial microRNA network elevates the threshold for infection-associated thymic involution via miR-29a mediated suppression of the IFN-alpha receptor. *Nat. Immunol.* 13 181–187. 10.1038/ni.2193 22179202 PMC3647613

[B40] Parkin J. D. San Antonio J. D. Pedchenko V. Hudson B. Jensen S. Savige J. (2011). Mapping structural landmarks, ligand binding sites, and missense mutations to the collagen IV heterotrimers predicts major functional domains, novel interactions, and variation in phenotypes in inherited diseases affecting basement membranes. *Hum. Mutat.* 32 127–143. 10.1002/humu.21401 21280145 PMC4800984

[B41] Peng D. W. Lan C. L. Dong L. Q. Jiang M. Xiao H. D’Amato R. (2022). Anti-angiogenic properties of microRNA-29a in preclinical ocular models. *Proc. Natl. Acad. Sci. U. S. A.* 119:e2204795119. 10.1073/pnas.2204795119 36322719 PMC9659377

[B42] Peng L. Li N. Huang Z. Qiu C. Yin S. (2022). Prognostic Gene Expression Signature for Age-Related Hearing Loss. *Front. Med.* 9:814851. 10.3389/fmed.2022.814851 35463035 PMC9021842

[B43] Peng Y. Song X. Zheng Y. Cheng H. Lai W. (2018). circCOL3A1-859267 regulates type I collagen expression by sponging miR-29c in human dermal fibroblasts. *Eur. J. Dermatol.* 28 613–620. 10.1684/ejd.2018.3397 30378537

[B44] Pozzi A. Yurchenco P. DIozzo R. V. (2017). The nature and biology of basement membranes. *Matrix Biol.* 5 1–11. 10.1016/j.matbio.2016.12.009 28040522 PMC5387862

[B45] Qiu F. Sun R. Deng N. Guo T. Cao Y. Yu Y. (2015). miR-29a/b enhances cell migration and invasion in nasopharyngeal carcinoma progression by regulating SPARC and COL3A1 gene expression. *PLoS One* 10:e0120969. 10.1371/journal.pone.0120969 25786138 PMC4364736

[B46] Rousset F. Nacher-Soler G. Coelho M. Ilmjarv S. Kokje V. Marteyn A. (2020). Redox activation of excitatory pathways in auditory neurons as mechanism of age-related hearing loss. *Redox. Biol.* 30:101434. 10.1016/j.redox.2020.101434 32000019 PMC7016250

[B47] Rudnicki A. Avraham K. B. (2012). microRNAs: the art of silencing in the ear. *EMBO Mol. Med.* 4 849–859. 10.1002/emmm.201100922 22745034 PMC3491818

[B48] Sakaguchi N. Spicer S. S. Thomopoulos G. N. Schulte B. (1997). Increased laminin deposition in capillaries of the stria vascularis of quiet-aged gerbils. *Hear. Res.* 105 44–56. 10.1016/s0378-5955(96)00180-3 9083803

[B49] Seicol B. J. Lin S. Xie R. (2022). Age-Related Hearing Loss Is Accompanied by Chronic Inflammation in the Cochlea and the Cochlear Nucleus. *Front. Aging Neurosci.* 14:846804. 10.3389/fnagi.2022.846804 35418849 PMC8995794

[B50] Semaan M. T. Zheng Q. Y. Han F. Zheng Y. Yu H. Heaphy J. (2013). Characterization of neuronal cell death in the spiral ganglia of a mouse model of endolymphatic hydrops. *Otol. Neurotol.* 34 559–569. 10.1097/MAO.0b013e3182868312 23462289 PMC3628741

[B51] Solda G. Robusto M. Primignani P. Castorina P. Benzoni E. Cesarani A. (2012). A novel mutation within the MIR96 gene causes non-syndromic inherited hearing loss in an Italian family by altering pre-miRNA processing. *Hum. Mol. Genet.* 21 577–585. 10.1093/hmg/ddr493 22038834 PMC3259013

[B52] Someya S. Prolla T. A. (2010). Mitochondrial oxidative damage and apoptosis in age-related hearing loss. *Mech. Ageing Dev.* 131 480–486. 10.1016/j.mad.2010.04.006 20434479 PMC4086639

[B53] Someya S. Yu W. Hallows W. C. Xu J. Vann J. Leeuwenburgh C. (2010). Sirt3 mediates reduction of oxidative damage and prevention of age-related hearing loss under caloric restriction. *Cell* 143 802–812. 10.1016/j.cell.2010.10.002 21094524 PMC3018849

[B54] Subramanian B. Kaya O. Pollak M. R. Yao G. Zhou J. (2018). Guided tissue organization and disease modeling in a kidney tubule array. *Biomaterials* 183 295–305. 10.1016/j.biomaterials.2018.07.059 30189357 PMC6671629

[B55] Sun G. Zheng Y. Fu X. Zhang W. Ren J. Ma S. (2022). Single-cell transcriptomic atlas of mouse cochlear aging. *Protein Cell*. 10.1093/procel/pwac058 [Epub ahead of print]. 36933008 PMC10098046

[B56] Tavanai E. Mohammadkhani G. (2017). Role of antioxidants in prevention of age-related hearing loss: a review of literature. *Eur. Arch. Otorhinolaryngol.* 274 1821–1834. 10.1007/s00405-016-4378-6 27858145

[B57] Ushakov K. Rudnicki A. Avraham K. B. (2013). MicroRNAs in sensorineural diseases of the ear. *Front. Mol. Neurosci.* 6:52. 10.3389/fnmol.2013.00052 24391537 PMC3870287

[B58] Wang Q. Zhao H. Zheng T. Wang W. Zhang X. Wang A. (2017). Otoprotective effects of mouse nerve growth factor in DBA/2J mice with early-onset progressive hearing loss. *J. Neurosci. Res.* 95 1937–1950. 10.1002/jnr.24056 28345280 PMC5561496

[B59] Weston M. D. Pierce M. L. Rocha-Sanchez S. Beisel K. Soukup G. (2006). MicroRNA gene expression in the mouse inner ear. *Brain Res.* 1111 95–104. 10.1016/j.brainres.2006.07.006 16904081

[B60] White H. J. Helwany M. Peterson D. C. (2022). *Anatomy, Head and Neck, Ear Organ of Corti StatPearls.* Treasure Island, FL: StatPearls. 30855919

[B61] Wilson S. E. Torricelli A. A. Marino G. K. (2020). Corneal epithelial basement membrane: Structure, function and regeneration. *Exp. Eye Res.* 194:108002. 10.1016/j.exer.2020.108002 32179076 PMC7217741

[B62] Wu P. Z. Liberman L. D. Bennett K. de Gruttola V. O’Malley J. Liberman M. (2019). Primary Neural Degeneration in the Human Cochlea: Evidence for Hidden Hearing Loss in the Aging Ear. *Neuroscience* 407 8–20. 10.1016/j.neuroscience.2018.07.053 30099118 PMC6369025

[B63] Zhang Q. Liu H. McGee J. Walsh E. Soukup G. He D. (2013). Identifying microRNAs involved in degeneration of the organ of corti during age-related hearing loss. *PLoS One* 8:e62786. 10.1371/journal.pone.0062786 23646144 PMC3640032

[B64] Zhao T. Liu X. Sun Z. Zhang J. Zhang X. Wang C. (2020). RNA-seq analysis of potential lncRNAs for age-related hearing loss in a mouse model. *Aging* 12 7491–7510. 10.18632/aging.103103 32335544 PMC7202524

[B65] Zhao T. Ma P. Zhao F. Zheng T. Yan B. Zhang Q. (2021). Phenotypic differences in the inner ears of CBA/CaJ and C57BL/6J mice carrying missense and single base pair deletion mutations in the Cdh23 gene. *J. Neurosci. Res.* 99 2743–2758. 10.1002/jnr.24905 34133797

[B66] Zheng Q. Y. Johnson K. R. Erway L. C. (1999). Assessment of hearing in 80 inbred strains of mice by ABR threshold analyses. *Hear. Res.* 130 94–107. 10.1016/s0378-5955(99)00003-9 10320101 PMC2855304

