# Supplementary material for: MiR-29a-deficiency causes thickening of the basilar membrane and age-related hearing loss by upregulating collagen IV and laminin
Source: Front Cell Neurosci. 2023 May 18;17:1191740. doi: 10.3389/fncel.2023.1191740 (PMC10232818; doi:10.3389/fncel.2023.1191740)
Supplement: Supplementary file 1 [file Table_1.DOCX]

**Supplementary Table 1**

Complementary sequences alignments of miR-29a seed regions with its binding sites in the 3'-untranslated region (3'-UTR) of *Col4a1*, *Col4a2*, *Col4a3*, *Col4a4*, *Col4a5*, *Lamb2* and *Lamc1*.

| **Target gene** | **Predicted binding sites** |
| --- | --- |
| *Col4a1* | 33-40 of Col4a1 3'-UTR 5'-GCCACCGUCACAACA**UGGUGCU**A  miR-29a-3p 3'-AUUGGCUAAAGUCU**ACCACGA**U |
| *Col4a2* | 43-49 of Col4a2 3'-UTR 5'-GUGGGAAGGCCACGU**UGGUGCU**U  miR-29a-3p 3'-AUUGGCUAAAGUCU**ACCACGA**U |
| *Col4a3* | 1752-1758 and 1776-1782 of Col4a3 3'-UTR  5'-UGGACGUGA**UGGUGCU**GAUACUUAAGUUCAGGC**UGGUGCU**GU  miR-29a-3p 3'-AUUGGCUAAAGUCU**ACCACGA**U |
| *Col4a4* | 89-95 of Col4a4 3'-UTR 5'-UUCCAGCAAAUUCAG**UGGUGCU**C  miR-29a-3p 3'-AUUGGCUAAAGUCU**ACCACGA**U |
| *Col4a5* | 129-135 of Col4a5 3'-UTR 5'-CACUGCUCCCACCAA**UGGUGCU**A  miR-29a-3p 3'-AUUGGCUAAAGUCU**ACCACGA**U |
| *Lamb2* | 2291-2297 of Lamb2 3'-UTR 5'-AGCCCCACGUCU**UGGUGCU**A  miR-29a-3p 3'-AUUGGCUAAAGUCU**ACCACGA**U |
| *Lamc1* | 1398-1404 of Lamc1 3'-UTR 5'-UAUUAUCUGACAC**UGGUGCU**A  miR-29a-3p 3'-AUUGGCUAAAGUCU**ACCACGA**U |

The red font indicates the binding site.
