# Supplementary material for: MiR-29a-deficiency causes thickening of the basilar membrane and age-related hearing loss by upregulating collagen IV and laminin
Source: Front Cell Neurosci. 2023 May 18;17:1191740. doi: 10.3389/fncel.2023.1191740 (PMC10232818; doi:10.3389/fncel.2023.1191740)
Supplement: Supplementary file 2 [file Table_2.DOCX]

**Supplementary Table 2**

Primer sequences used for qRT-PCR.

| **Gene** | **Forward (5**'**-3**'**)** | **Reserve (5**'**-3**'**)** |
| --- | --- | --- |
| *Col4a1* | TCATTAGCAGGTGTGCGGTT | GCAGAGGCGAGCATCATAGT |
| *Col4a2* | CCCGGATCTGTACAAGGGTG | CGCCTTTTGAGATTACGCCG |
| *Col4a3* | GATGGGCTATCCTGGAACCACT | TTCTCTCCTCGTTCGCCTTTGG |
| *Col4a4* | GAACCTGGAAGAAAGGGAGAGG | GGAAGTGACTGCTTCTCCTGCA |
| *Col4a5* | GAACCTGGTCCTGTAGGTGC | TCTCCCAGGAGGGCCTTG |
| *Lamb2* | TACCCACACGGTCGGGAT | ACCATTGGAACCCCGTTCTC |
| *Lamc1* | CTTCATCGCCCCTGTGAAGT | CGTCTCACAGAACTGTCCCC |
| *Gapdh* | AGACAGCCGCATCTTCTTGT | CTTGCCGTGGGTAGAGTCAT |
