# Supplementary figures and images for: MiR-29a-deficiency causes thickening of the basilar membrane and age-related hearing loss by upregulating collagen IV and laminin
Source: Front Cell Neurosci. 2023 May 18;17:1191740. doi: 10.3389/fncel.2023.1191740 (PMC10232818; doi:10.3389/fncel.2023.1191740)

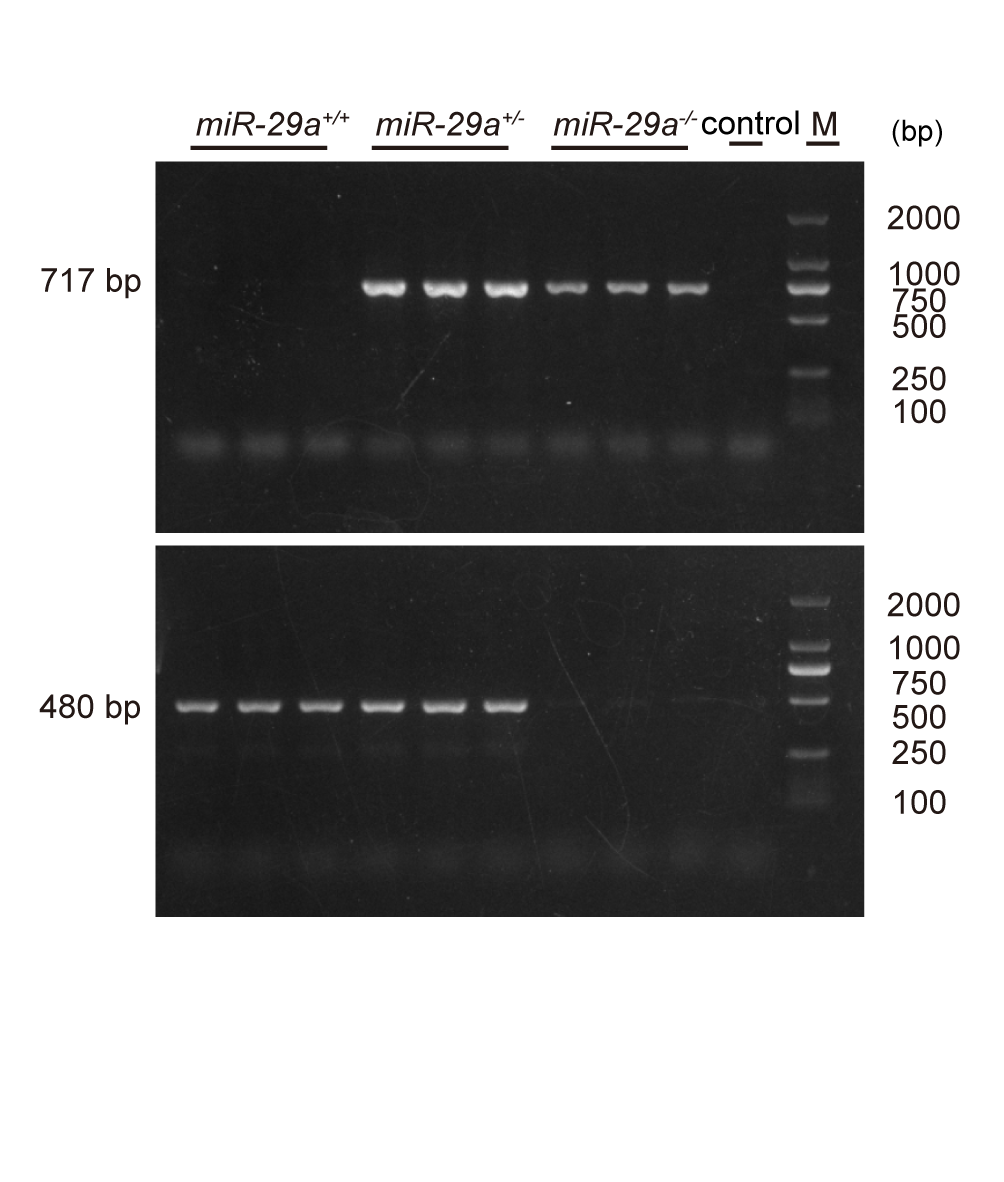

Supplement: Supplementary file 3 [file Image_1.TIF]

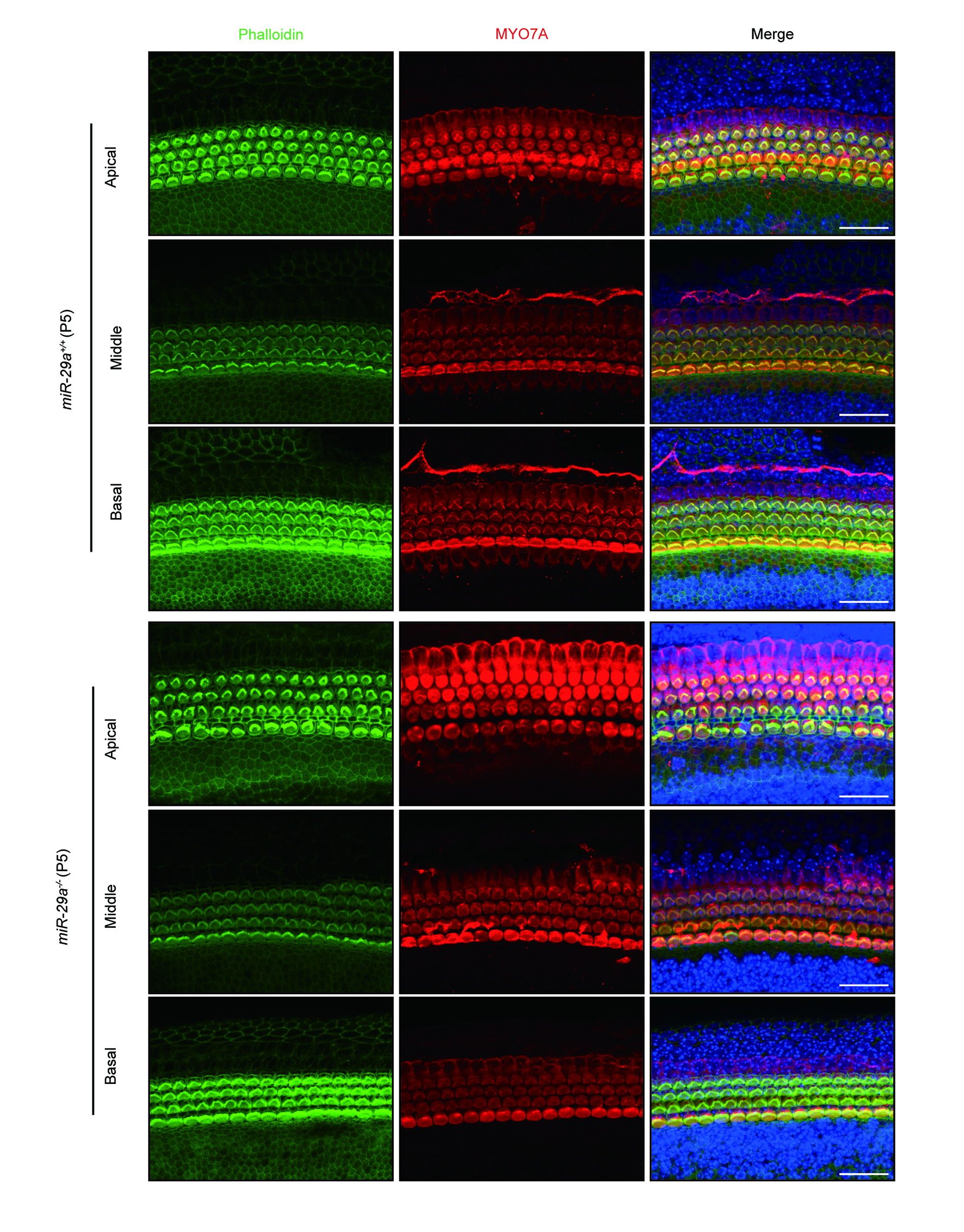

Supplement: Supplementary file 4 [file Image_2.TIF]
